# Electromagnetic field stimulation modulates working memory and cortical alpha oscillations in healthy adults

**DOI:** 10.1038/s41598-026-42063-4

**Published:** 2026-02-26

**Authors:** Kate S. Branigan, Kevin S. Saroka, Paula L. Corradini, Michel A. Larivière, Blake T. Dotta

**Affiliations:** 1 Behavioural Neuroscience Programs, Schools of Natural Science and Kinesiology & Health Sciences, Laurentian University, Sudbury, ON P3E2C6 Canada; 2 Biology Programs, Schools of Natural Science and Kinesiology & Health Sciences, Laurentian University, Sudbury, ON P3E2C6 Canada; 3 Human Kinetics Programs, Schools of Natural Science and Kinesiology & Health Sciences, Laurentian University, Sudbury, ON P3E2C6 Canada

**Keywords:** electromagnetic fields (EMF), electroencephalography (EEG), WAIS-IV, working memory, neural modulation, Neuroscience, Cognitive neuroscience, Learning and memory

## Abstract

Electromagnetic fields (EMFs) have been shown to modulate neural activity, yet their specific effects on memory, particularly the distinction between working memory and short-term recall, remain unresolved. This study examined whether patterned EMF exposure influences these domains, using the WAIS-IV Digit Span Forward (DSF) and Digit Span Backward (DSB) subtests. Ninety-eight healthy volunteers were randomly assigned, in a between-subjects design, to one of four conditions: Theta-Burst (five-pulse bursts at 100 Hz), Theta-Gamma (mimicking theta–gamma coupling in hippocampal networks), 40 Hz gamma stimulation, or sham stimulation. Fields were applied for 30 min in one of three spatial configurations: unilaterally over the left hemisphere, unilaterally over the right hemisphere, or bilaterally over the temporal lobes. Theta-Burst EMF reduced working memory performance on the DSB task and was accompanied by increased high-alpha (10–12 Hz) activity in the left inferior frontal gyrus and a whole-brain effect centered on the right superior frontal gyrus. In contrast, Theta-Gamma EMF reduced DSF performance without detectable EEG changes. These findings indicate that EMF effects on memory are frequency- and pattern-specific, selectively altering behavior and, in some cases, underlying neural activity. Optimizing stimulation parameters may allow such fields to be harnessed for cognitive enhancement or targeted neuromodulation.

**Supplementary Information:**

The online version contains supplementary material available at 10.1038/s41598-026-42063-4.

## Introduction

Cognition is dynamic, with neural processes constantly adapting to both internal and external influences. Emerging evidence suggests that electromagnetic fields (EMFs) function as a non-invasive neuromodulatory tool capable of altering neural oscillations and cognitive performance^1,2^. Understanding how EMFs interact with brain dynamics is crucial for both foundational neuroscience and potential clinical applications.

Working memory—the ability to temporarily store and manipulate information for cognitive tasks—is a core component of cognitive function^3,4^. Higher-frequency alpha activity, particularly within the 10–12 Hz range, is increasingly recognized as playing a key role in attentional regulation, cognitive control, and memory-related processes. High alpha oscillations are thought to reflect top-down inhibitory mechanisms that help suppress irrelevant information and optimize neural efficiency during cognitive tasks^5–7^. However, dysregulation of high alpha activity, particularly in frontal and parietal regions, has been linked to poorer working and short-term memory performance^8–11^. Suggesting that excessive inhibitory signaling may disrupt task-relevant processing. Given the importance of alpha rhythms in shaping functional connectivity within frontoparietal and default mode networks, examining how EMFs modulate high alpha activity may shed light on their influence over cognitive dynamics, information processing, and their potential relevance to neurocognitive disorders.

EMFs, characterized by dynamic forces, are a pervasive aspect of modern environments, spanning natural sources such as the Earth’s geomagnetic field to artificial exposures from technological devices^12–14^. The potential impact of EMFs on biological systems, particularly the central nervous system, has drawn increasing attention, with evidence suggesting that EMF effects are highly dependent on field intensity, frequency, and exposure pattern^15–22^. Rather than exerting uniform effects, EMFs appear to act as structured modulatory inputs, influencing biological processes in ways that are often nonlinear and parameter-specific. For instance, a systematic review on EMF effects in rodent spatial memory^23^ revealed inconsistent outcomes, highlighting the necessity of precise EMF patterning—an effect akin to pharmacological dose-response relationships. This variability emphasizes the need for controlled investigations into how EMFs influence cognitive processes across species and experimental conditions.

Prior research has shown that patterned EMF exposure can influence cognitive processes, including spatial learning in rodents^23^, working memory performance in humans^24–29^, and long-term synaptic plasticity in hippocampal slice preparations^30,31^. Expanding on these findings, research from our lab has demonstrated that specific EMF patterns induce targeted biological effects, including learning effects in flatworms^32^ and the facilitation of flow states in human participants^33^. These results align with broader work suggesting that the influence of EMFs extends beyond simple exposure effects, potentially modulating neurophysiological states in a controlled manner. Ghassemkhani et al.^32^ showed that Theta-Burst EMFs impair learning in planaria, suggesting a direct interaction between patterned EMFs and fundamental learning processes. Yoshikawa and Tateno (2025) extended this line of evidence to the mammalian cortex, demonstrating that localized theta-burst magnetic stimulation can bidirectionally modulate neural activity in the mouse auditory cortex in vivo, further supporting the capacity of patterned EMFs to induce targeted, frequency-specific changes in brain function^34^. These studies align with research on the neuromodulatory potential of Theta Burst stimulation. Li et al. (2014) demonstrated that prefrontal Theta Burst transcranial stimulation significantly altered cortical excitability and depressive symptoms in a randomized, sham-controlled study^24^. Together, these findings reinforce the idea that specific EMFs can meaningfully modulate brain function, suggesting their relevance in broader cognitive and behavioral processes, including memory and attentional control. More broadly, they support the view that EMFs are not merely passive environmental stimuli but active neuromodulatory forces capable of shaping neural dynamics and cognitive states.

The present study builds on this framework, investigating whether controlled EMF exposure can systematically alter short-term memory and frontoparietal network activity, with a specific focus on Theta Burst patterning as a potential driver of cognitive modulation. We tested whether controlled EMF exposure modulates short-term memory and frontoparietal network activity using three distinct field patterns: Theta-Gamma (mimicking theta-gamma coupling within hippocampal networks), Theta-Burst (five-pulsed bursts at 100 Hz with alternating amplitudes), and 40 Hz gamma stimulation. These fields were applied at different spatial configurations to examine their differential effects on working memory and EEG-based neural dynamics. While substantial evidence links EMFs to neural activity, few studies have thoroughly examined their influence on short-term memory and working memory dynamics in human subjects. Recent studies across animal and human models have shown that patterned EMF or magnetic stimulation can bidirectionally modulate cortical activity^34^, enhance cognitive performance^35,36^, and improve memory in clinical populations through gamma-frequency stimulation^28^. By integrating behavioral and electrophysiological data, we assess the immediate modulatory effects of Theta Burst EMFs on EEG dynamics, focusing on regions central to working memory, attentional processing, and frontoparietal network coordination. Understanding how EMFs modulate neural oscillations and memory could inform both fundamental neuroscience and translational efforts in cognitive enhancement and therapeutic interventions.

## Methods

### Participants

A total of 100 volunteers were recruited for participation in the study; however only 98 subject’s WAIS-IV scores were utilized for analysis. Two participants WAIS-IV were omitted from the dataset as they had performed the assessment within the last year. Each participant was randomly assigned a condition (control or EMF exposure group), those who were subjected to EMF exposure were randomly assigned an EMF condition. Average age of participants was 23.99 ± 5.45 years (range: 19–39). All participants had completed at least a high school diploma; most participants were university or college students (gender distribution: 35 male, 65 female). No formal a priori power analysis was performed, as reliable and consistent effect size estimates for patterned electromagnetic field modulation of working memory were not available at the time of study design. The target sample size (*N* = 100) was therefore determined a priori based on feasibility and consistency with prior human EMF and neuromodulation studies using comparable behavioral and electrophysiological outcomes. Analyses of secondary behavioral outcomes were treated as exploratory, and effect sizes are reported for all behavioral analyses to facilitate interpretation independent of null-hypothesis significance testing.

### Wechsler adult intelligence scale

The working memory and cognitive functioning of participants was assessed utilized three subtests of the Wechsler Adult Intelligence Scale, 4th edition (WAIS-IV). The three tests used were Digit Span (Forward, Backward, and Sequencing), Arithmetic, and Letter-Number Sequencing, which were assessed in that order. Each score was standardized, and age matched, and then compared to a normalized data set. This resulted in seven standardized scores assigned to each participant: Digit Span Forward (DSF), Digit Span Backward (DSB), Digit Span Sequencing (DSS), Digit Span Total (DST), Arithmetic (ARITH), Letter-Number Sequencing (LNS), and Working Memory Index (WMI). DST is a composite score of DSF, DSB, and DSS. The WMI is another composite score of DST and ARITH. Lower values in these composite scores indicated worse cognitive performance. The qualifications for participants to undergo WAIS-IV assessments was that they were over the age of 18 and had not performed the task in the last year, and majority of participants had never taken WAIS-IV assessments.

### EMF protocol

**Fig. 1:**
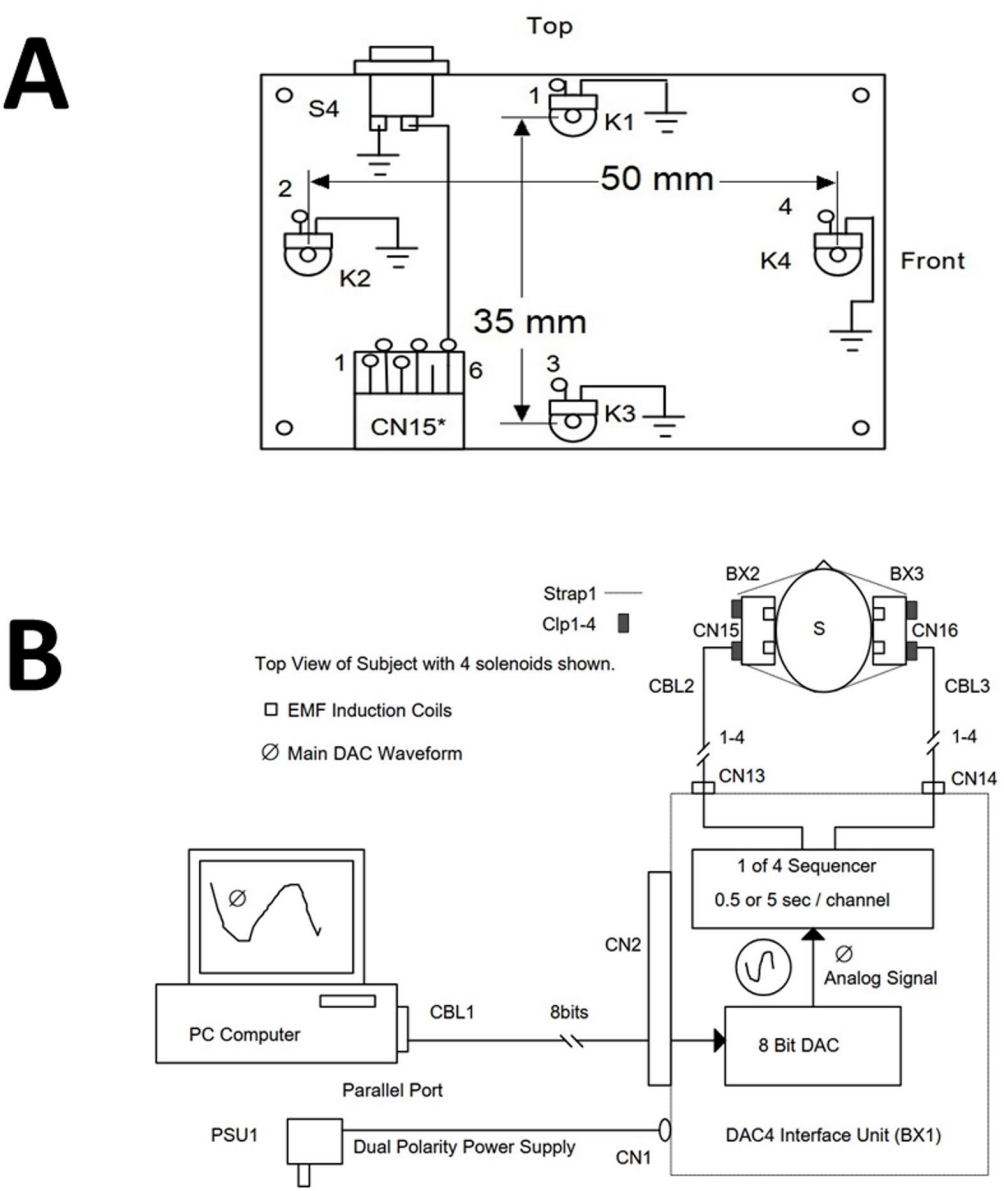
**(A)** The solenoid array measuring 50 mm along the horizontal axis and 35 mm along the vertical axis. Each box is centered over T3 and T4 EEG landmarks. The left and right sides have opposite magnetic polarity as illustrated and solenoid pair 4 is oriented towards the front of head. (**B**) Block Diagram of SAM-360 Interface Unit and Solenoid Arrays. The Solenoids are 250 Ohms with a maximum power output 100 mW. Radio Shack InterTan 275 − 232 5VDC Reed Relay Reed Switch with core removed and replaced with a steel flux concentrator.

The EMF was generated and applied using two solenoids (8.2 cm × 3.4 cm × 5.3 cm) affixed to participants’ heads via a headband. Participants were randomly assigned to one of three EMF exposure conditions: Theta-Gamma, which mimicked theta-gamma coupling within hippocampal networks; Theta-Burst, consisting of five-pulsed bursts at 100 Hz with alternating amplitudes; or 40 Hz stimulation, a classical gamma-frequency field. The EMF patterns were stored on a Zenith computer and configured using a customized software program, Complex, which controlled the signal transmission via a digital-to-analog converter (DAC). The electrical pattern was then transmitted to the solenoids, generating a corresponding magnetic field at a strength of approximately 4 µT; a detailed description of the generation of the magnetic field, irrespective of pattern, has been previously described^13^ and illustrated in the block diagram observed in Fig. 1. To examine the spatial specificity of the EMF effects, fields were applied in one of three configurations: bilateral left (left frontal and left temporal lobes), bilateral right (right frontal and right temporal lobes), or bilateral temporal (left and right temporal lobes). These configurations were chosen to assess whether EMF exposure could differentially influence brain regions involved in memory encoding and retrieval. Each exposure session lasted 30 min, immediately preceding cognitive and electrophysiological assessments. In the sham condition, solenoids were mounted in identical positions and for the same duration as in the active stimulation sessions, but no electromagnetic field was generated.

### Procedure

**Fig. 2:**
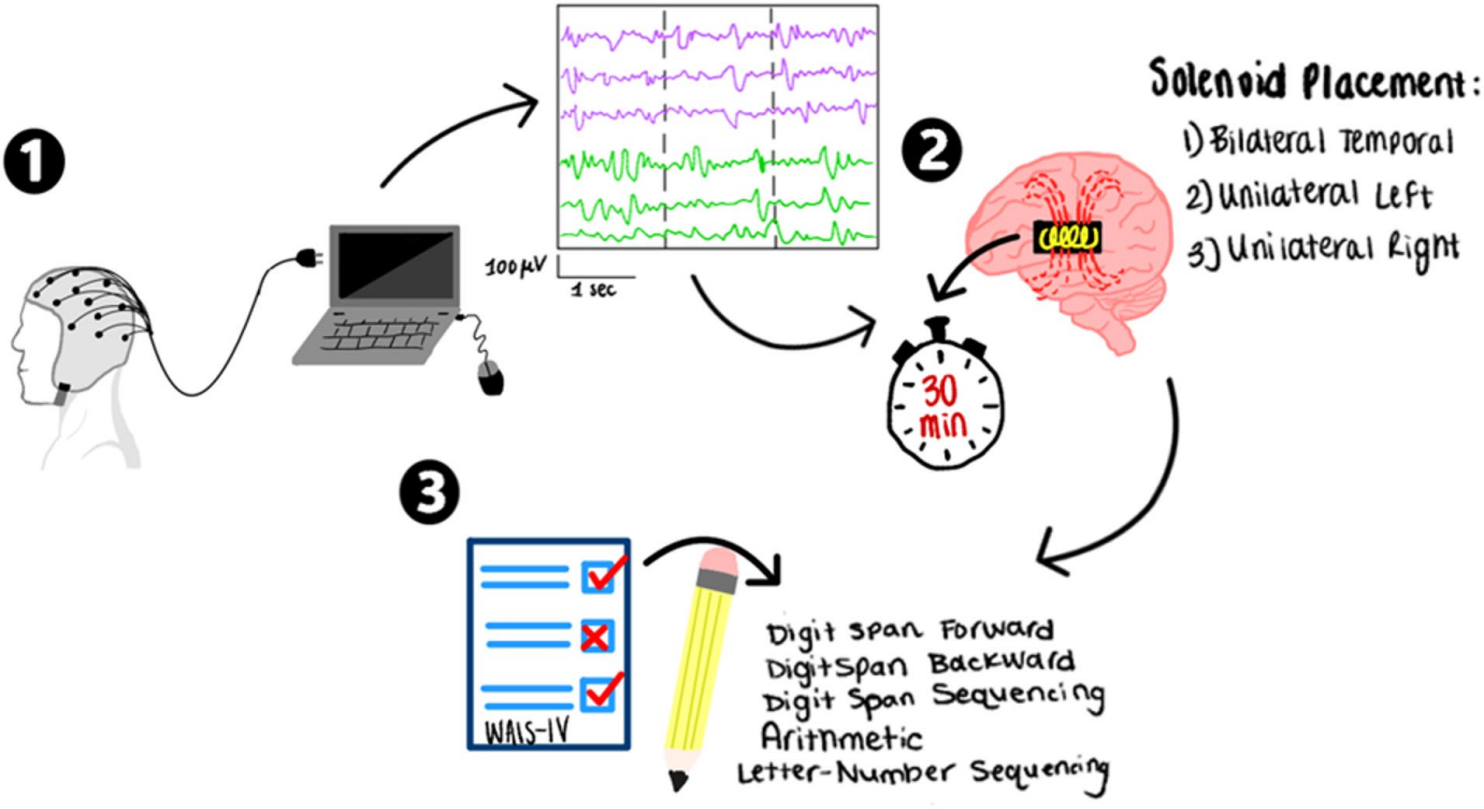
Visual representation of the experimental procedure. Participants were fitted with an EEG cap and their brain activity was recorded continuously (1), solenoids were placed in one of three possible placements and EMF was exposed for 30 min (2), finally participants were asked to perform specific subtests of the WAIS-IV (3). See Supplementary Figure S1 for a detailed procedural flowchart.

The current experiment was reviewed and approved by the Research Ethics Board of Laurentian University (file no. 6021235). All methods were performed in accordance with the relevant guidelines and regulations. Informed consent was obtained from all participants prior to participation. Trials were conducted in a sound-proof room in the Laurentian University Consciousness Lab. Informed consent as approved by the Research Ethics Board of Laurentian was obtained prior to each experiment. Each participant was randomly assigned to one of four experimental groups prior to the initiation of the experiment. An EEG cap was applied to the head of each participant prior to the beginning of the experiment. EMF-generating solenoids were applied to the head. Both prior to and following the experiment, participants were instructed to open their eyes for 2 min and then close their eyes for 2 min. These recordings were done to provide a measure of baseline cortical activity. Following the initial baseline measurements, one of the three stories was read and then participants were exposed to 30 min of an EMF, during which they were instructed to keep their eyes open. After completion of the EMF exposure, the evaluator would enter the room with the participant and administer the cognitive assessments from the WAIS-IV (Digit Span, Arithmetic, and Letter-Number Sequencing). Following completion of cognitive assessments, participants were instructed to keep their eyes open for 2 min and then close their eyes for 2 min. The experiment was concluded when the participant was presented with a debriefing form and detached from equipment. A visual representation of the experimental procedure can be seen in Fig. 2.

### EEG collection and processing

Electrical activity of the brain was monitored and measured using a Mitsar 201 amplifier, equipped with a 19-channel electroencephalography (EEG) cap. Measurements from 19 sites (Fp1, Fp2, F7, F3, Fz, F4, F8, T3, C3, Cz, C4, T4, T5, P3, Pz, P4, T6, O1, O2) that were linked to the ears (A1 and A2) for monopolar recordings, following the 10–20 International Standard of Electrode Placement were recorded. The data was recorded on a Lenovo laptop with WinEEG, which produced a digital copy of the recorded voltages. The data collected were recorded using a sampling rate of 250 Hz. The data were artifact corrected in WinEEG using individual component analysis (ICA) to correct for eye blinks which were observed as transient spikes (< 1 Hz oscillations) over the Fp1 and Fp2 sensors. Following ICA, 10-second excerpts were taken from the baseline measurements, both pre- and post-exposure, and exported as text files. The text files were then imported to sLORETA for spectral analysis.

The Utilities application in LORETA was utilized to convert text files into sLORETA-compatible files. The electrode positions were converted into coordinates through the Electrode names to coordinates function. Subsequently, the electrode coordinates were inputted into the Electrode coordinates to transformation matrix function, where they were converted into a transformation matrix based upon the 3D profile of the human cerebrum. The text files containing EEG data were then imported to EEGs to cross spectrum in which number of electrodes (*N* = 19), number of time frames per epoch (in this case, 2500), and sampling rate were also added. Computation was completed across the classical frequency bands (delta, theta, alpha1, alpha2, beta1, beta2, beta3, and gamma). The resulting cross spectrum files in addition to the computed transformation matrix were then imported to Cross spectra to sLORETA, where they were computed into cross-spectral densities for analysis. A standard sLORETA analysis was performed in which the raw qEEG extractions were converted into cross-spectral and then sLORETA documents.

Source projection using sLORETA was not employed to achieve precise spatial localization, which is inherently limited with low-density EEG and in the absence of individual anatomical information. Instead, source projection was used as a spatial regularization and dimensional-reduction approach to summarize oscillatory activity within anatomically constrained regions and to reduce reference-dependent and volume-conduction effects present at the sensor level. All source-level results are therefore interpreted conservatively at the regional level and not as fine-grained anatomical localization.

### Data analysis

Performance on WAIS-IV tests was analyzed using SPSS within a hierarchical statistical framework reflecting the study’s a priori hypotheses. The comparison between the Theta Burst EMF condition and Sham was specified as the primary confirmatory behavioral hypothesis, based on prior evidence indicating that Theta Burst stimulation disrupts working memory performance. Accordingly, standardized WAIS-IV scores were compared between the Theta Burst and Sham conditions using planned, hypothesis-driven tests. In contrast, the Theta–Gamma and 40 Hz EMF conditions were included to examine potential effects of alternative stimulation patterns for which no established behavioral literature exists and were therefore treated as exploratory. Analyses involving these conditions were evaluated using omnibus one- and two-way analyses of variance (ANOVAs), with EMF exposure pattern as the independent variable. Where omnibus effects were observed, post hoc comparisons were performed using Tukey’s HSD to account for multiple pairwise comparisons. Effect sizes were quantified using Cohen’s d for pairwise contrasts and partial η² for omnibus effects. Omnibus ANOVA results are reported to provide context for the overall pattern of effects but were not treated as a prerequisite for evaluating planned contrasts associated with the primary hypothesis. Given the unequal gender distribution in the sample (35 males, 65 females), supplementary analyses were conducted in which gender was examined as a between-subjects factor and as a covariate to assess potential confounding effects on behavioral outcomes.

To characterize potential EMF-related changes in cortical oscillatory activity, an initial whole-brain analysis was conducted using a non-parametric statistical framework. Independent-group comparisons across the four EMF conditions were performed using statistical non-parametric mapping (SnPM), with empirical null distributions generated from 5,000 random permutations of group labels. Statistical significance was assessed using a maximum-statistic approach across all voxels within each frequency band, thereby controlling the family-wise error rate for multiple comparisons.

This whole-brain analysis was used to evaluate whether any global or regionally distributed effects of EMF exposure were present and to characterize their general topography. It was not used to define regions, contrasts, or statistical thresholds for confirmatory hypothesis testing. Based on a priori hypotheses regarding working memory networks, region-of-interest (ROI) analyses were subsequently conducted focusing on the inferior frontal gyrus (IFG) and dorsal inferior parietal cortex (DIPC), independent of the whole-brain results. In addition, a frontal region exhibiting a maximal whole-brain effect was examined descriptively. ROI time series were extracted across eight canonical frequency bands: delta (0.5–4 Hz), theta (4–8 Hz), low alpha (8–10 Hz), high alpha (10–12 Hz), beta-1 (13–20 Hz), beta-2 (20–25 Hz), beta-3 (25–30 Hz), and gamma (> 30 Hz), with seed coordinates listed in Table 1. Extracted cross-spectral density measures were then used for subsequent statistical analyses.

Given the limited existing literature describing the electrophysiological consequences of patterned EMF exposure in humans, the whole-brain SnPM analysis was intended to provide an unbiased characterization of whether EMF-related oscillatory effects were present and to identify their general cortical distribution. Separately, and prior to data analysis, the IFG and DIPC were selected as a priori regions of interest based on their critical involvement in working memory and related executive processes that are susceptible to neuromodulatory influences, particularly those delivered at theta frequencies^37–40^. The IFG is a core component of large-scale executive and attentional control networks, supporting inhibitory control, interference resolution, and flexible updating of task-relevant information^41,42^. The DIPC, encompassing the intraparietal sulcus and surrounding inferior parietal regions, plays a central role in the maintenance and manipulation of information in working memory, attentional shifting, and the integration of multimodal sensory inputs^43–45^. Both regions are integral nodes of the fronto-parietal control network, which coordinates goal-directed behavior and adapts cognitive strategies in response to task demands. Prior work has shown that oscillatory activity in the theta and high-alpha ranges facilitates long-range communication between frontal and parietal cortices during memory encoding, retrieval, and executive control^46^. These regions were therefore targeted for hypothesis-driven ROI analyses independent of the whole-brain results. The observation that maximal whole-brain effects were frontal was considered descriptive and consistent with these hypotheses, but did not determine ROI selection or statistical testing.

**Table 1:** Regions-of-interest (ROIs) and their associated MNI coordinates utilized in the statistical analyses.

Region	X	Y	Z
ROIs Identified from multiple comparisons within the ‘Statistics’ function of LORETA software			
R Superior Frontal Gyrus	20	65	-15
ROIs extracted for exploratory effects implicated in working memory			
Left DIPC	-50	-55	45
Right DIPC	45	-45	40
Left Inferior Frontal G.	-35	25	0
Right Inferior Frontal G.	35	25	0

## Results

### Primary WAIS-IV Analysis: Theta Burst EMF vs. Control

**Fig. 3:**
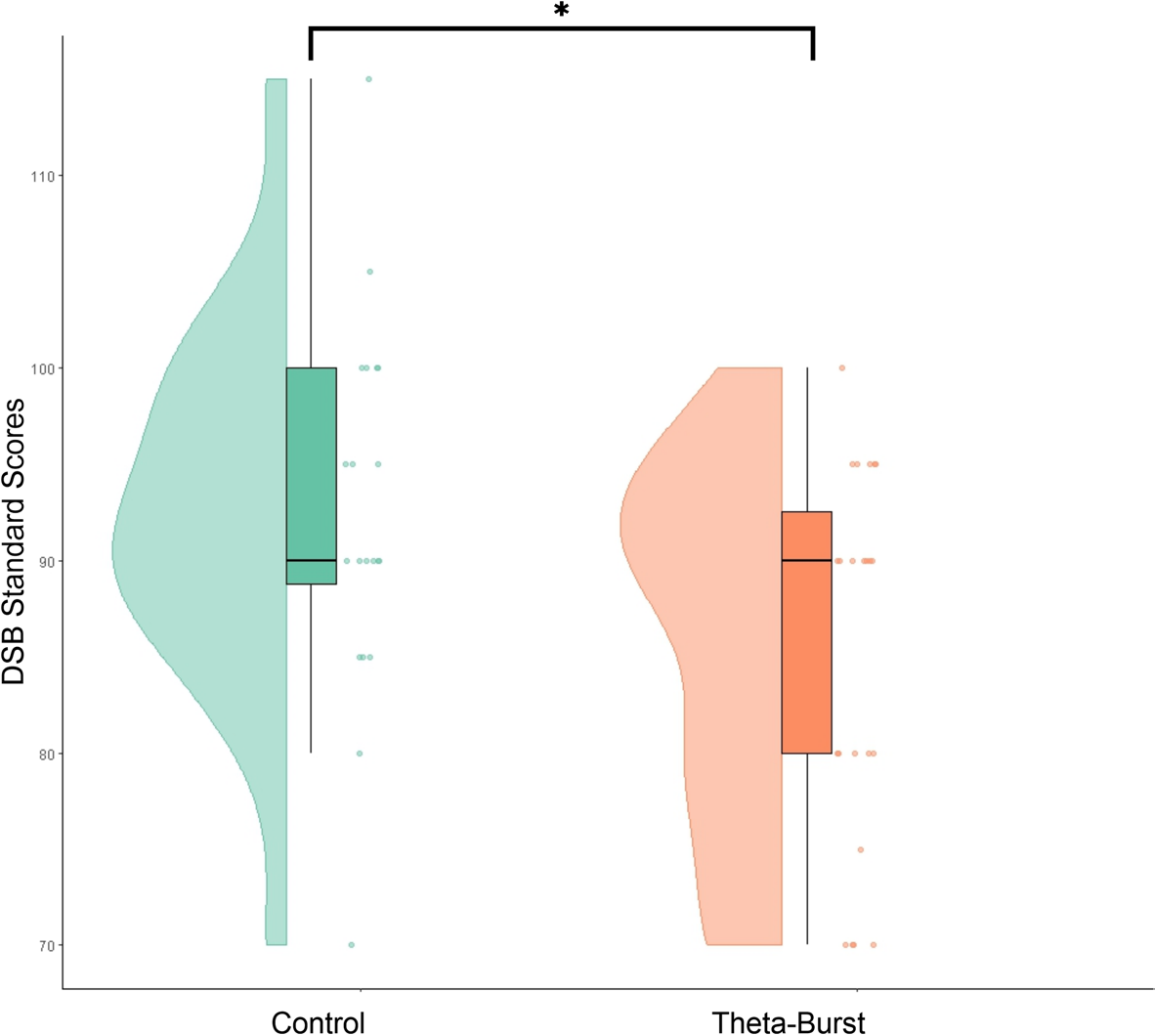
Raincloud plot of Digit Span Backward (DSB) standard scores across sham and Theta Burst EMF. Participants exposed to Theta Burst EMF exhibited a significant reduction in DSB performance compared to the Control condition (*p* = 0.018), indicating a suppressive effect on working memory. Raincloud plots display individual data points (right), boxplots indicating the interquartile range with whiskers representing the data range (center), and half-violin plots illustrating the distribution density (left).

Planned t-tests were conducted based on an a priori hypothesis that Theta Burst EMFs would negatively affect memory performance. Given this directional expectation, five WAIS subtests were analyzed immediately following exposure. Theta Burst EMFs significantly reduced DSB scores compared to the Control condition [t(41) = 2.47, *p* = 0.018, Cohen’s d = 0.76], indicating a specific suppressive effect on working memory. Participants exposed to Theta Burst EMFs demonstrated significantly lower DSB performance compared to the Sham condition [Sham: 92.5(9.66) vs. Theta Burst: 85.21(9.5), Mean ± SD], supporting the hypothesis that Theta Burst EMF impairs working memory. These findings suggest that Theta Burst EMF exposure may alter cognitive processing in a targeted manner, consistent with prior research on Theta Burst stimulation. This result is shown in Fig. 3.

No statistically significant main effects of gender or gender × condition interactions were observed for any behavioral measure, and inclusion of gender in the models did not alter the direction or significance of the primary condition effects.

### Exploratory WAIS-IV Analysis: Theta Gamma & 40 Hz EMFs vs. Control

**Fig. 4:**
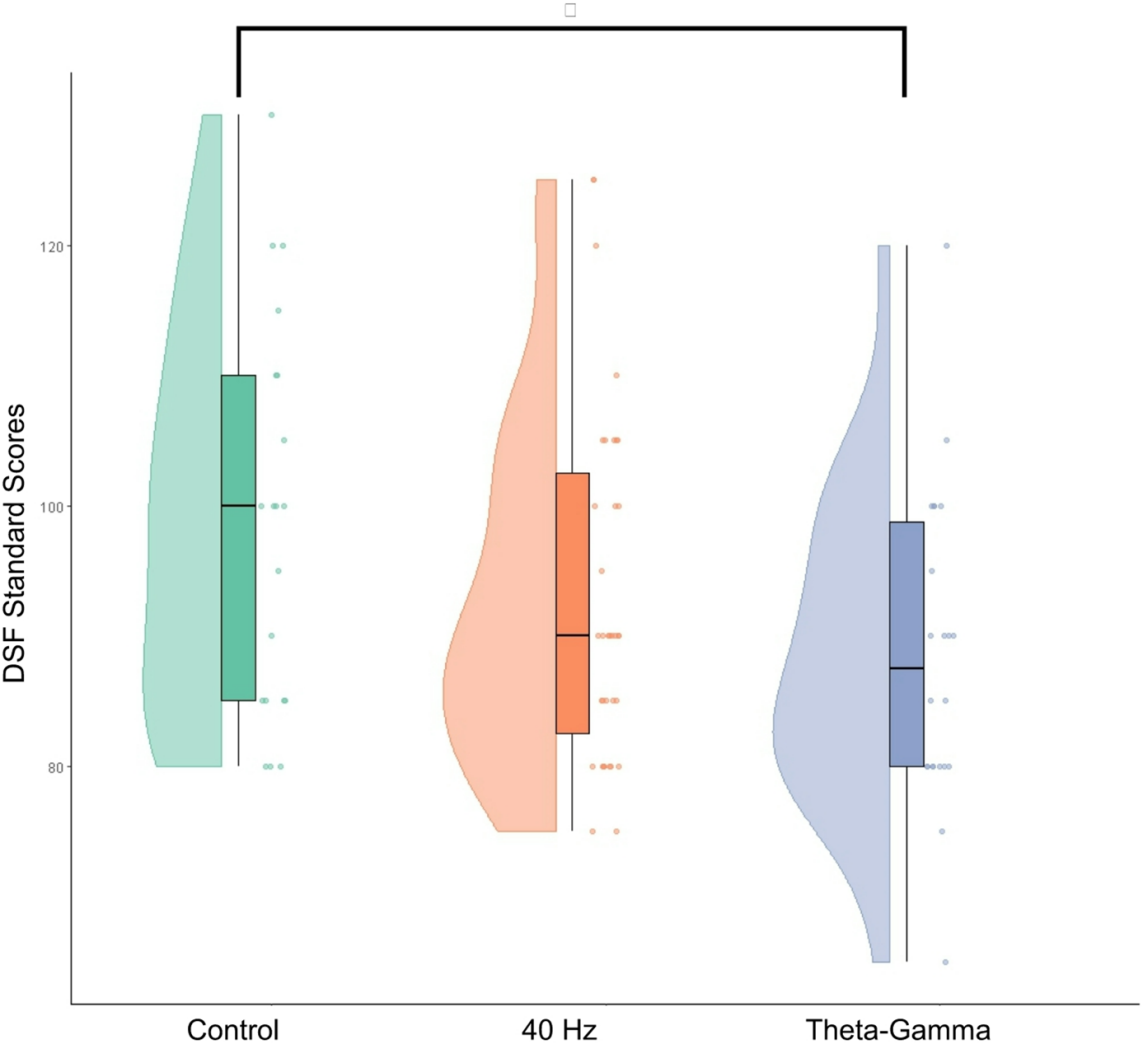
Raincloud plots of Digit Span Forward (DSF) scores across experimental conditions. Participants exposed to Theta Gamma EMF exhibited a reduction in DSF performance compared to the Sham condition, with a significant effect observed in post hoc comparisons (*p* = 0.047, Cohen’s d = 0.74). Raincloud plots display individual data points (right), boxplots indicating the interquartile range with whiskers representing the data range (center), and half-violin plots illustrating the distribution density (left). The asterisk (*) denotes a significant difference.

A one-way ANOVA was conducted on WAIS-IV scores for the Theta–Gamma and 40 Hz EMF conditions to examine potential effects on short-term memory performance. For DSF scores, the omnibus test did not reach statistical significance [F(2,74) = 2.9, *p* = 0.06, partial η²=0.09]. Consistent with our a priori analytical criteria, post hoc comparisons were therefore examined for descriptive and hypothesis-generating purposes only. A Tukey HSD comparison indicated lower DSF scores in the Theta–Gamma EMF condition relative to Sham (*p* = 0.047, Cohen’s d = 0.74) [Sham: 98.8 (15.12) vs. Theta–Gamma: 88.63 (12.16), Mean ± SD]. Because the omnibus criterion for confirmatory inference was not met, this effect is interpreted as exploratory rather than confirmatory. This can be seen in Fig. 4.

Descriptive statistics for WAIS-IV subtest and composite scores across the four EMF exposure conditions are presented in Table 2. Across all subtests, mean standardized scores were within the expected normative range for each group. Demographic characteristics were similar across groups, with mean ages ranging from 23.37 to 24.99 years. Sex ratios (M: F) varied modestly, and group sizes ranged from 20 to 36 participants.

**Table 2:** Mean (± SD) WAIS-IV subtest and composite scores by EMF exposure condition.

Condition	Control	40 Hz	Theta-Gamma	Theta-Burst
DSF	98.75 (± 14.74)	93 (± 13.26)	88.64 (± 11.88)	92.17 (± 13.66)
DSB	92.5 (± 9.42)	88.57 (± 11.12)	87.73 (± 7.65)	85.23 (± 9.38)
DSS	88 (± 10.59)	86.43 (± 12.85)	88.18 (± 11.13)	84.35 (± 10.56)
DST	90.5 (± 12.03)	86.43 (± 11.74)	83.64 (± 11.09)	84.56 (± 11.69)
ARITH	91 (± 13.28	91.14 (± 15.21)	92.95 (± 10.52)	89.13 (± 14.50)
LNS	95.25 (± 11.21)	91.14 (± 12.65)	88.64 (± 9.79)	88.04 (± 15.79)
WMI	88.7 (± 13.25)	86.57 (± 13.85)	85.91 (± 10.03)	84 (± 14.58)
AGE	24.14 (± 5.39)	23.37 (± 3.70)	23.83 (± 6.17)	24.99 (± 6.76)
M: F	6:14	15:21	9:13	6:17
Group size (N)	20	36	22	23

### Exploratory EEG Analysis: EMF Conditions vs. Control

**Fig. 5:**
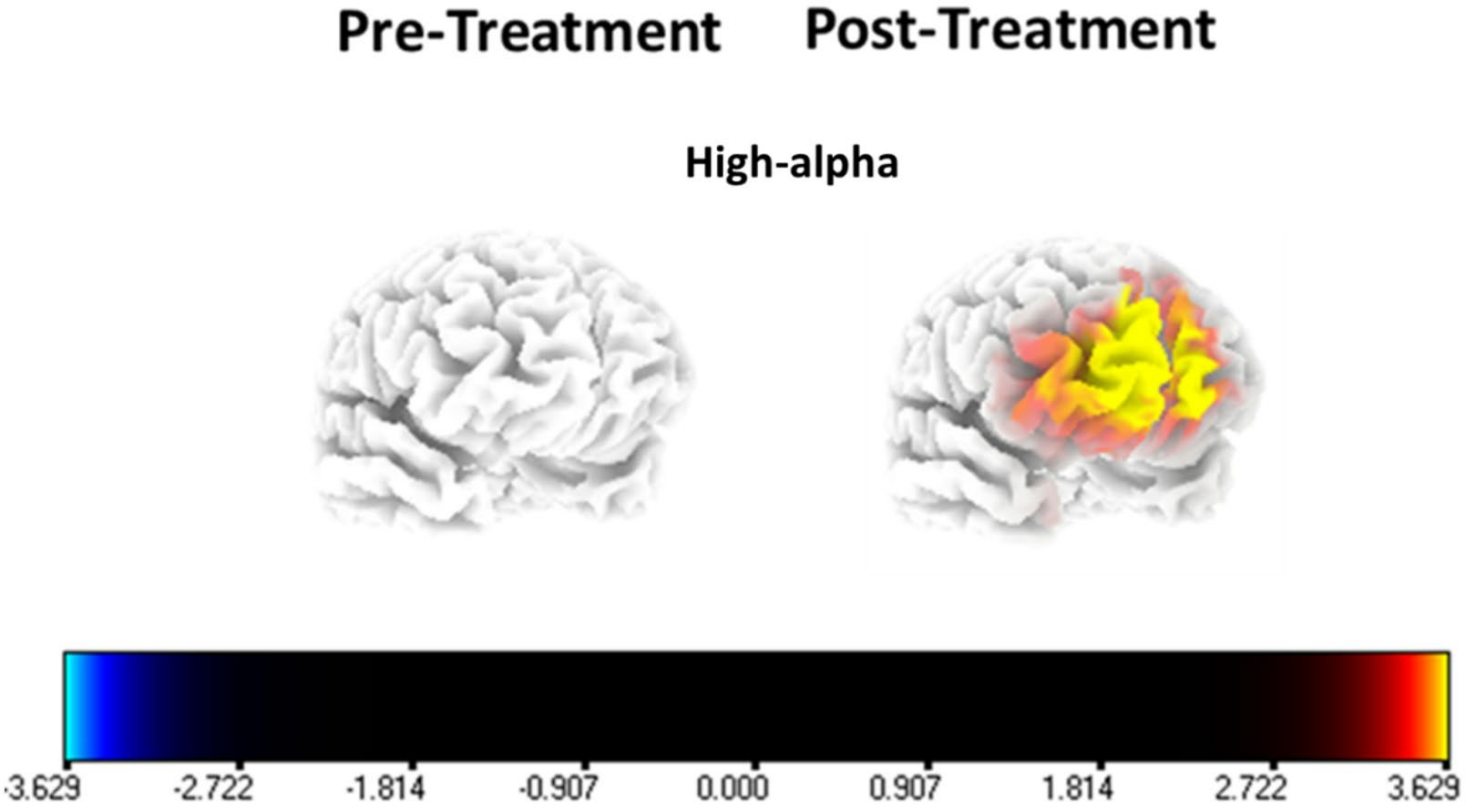
Exposure to the Theta-Burst EMF pattern significantly increased high-alpha (10–12 Hz) activity in the right superior frontal gyrus, extending into bilateral medial frontal regions (*p* = 0.032). All source localization was completed using LORETA software v. 20,240,713^48^, downloaded from https://www.uzh.ch/keyinst/loreta which comes packaged with Utilities and an Explorer for analyzing the results of source localization as well as generating images.

We employed the LORETA statistical packages to discern potential effects of the three EMF field patterns on eyes-closed brain electrical activity recorded before and after the exposure period. All data were log-transformed before t-tests were performed. No significant between-group differences were observed at baseline. Post-treatment, participants exposed to the Theta-Burst pattern exhibited significantly greater high-alpha (10–12 Hz) power, with a regional maximum centered in the right superior frontal gyrus (MNI: 20, 65, − 15; t = 3.91, *p* = 0.032), extending into inferior and medial frontal regions bilaterally. This result can be seen in Fig. 5.

For the exploratory ROI analysis, we first computed percentage change (relative) scores for each ROI. The scores were calculated as follows:

$$Relative\%=100\times\left(\frac{\left(\mathrm{E}\mathrm{C}2\right)-\left(EC1\right)}{\left(EC1\right)}\right)$$

**Fig. 6:**
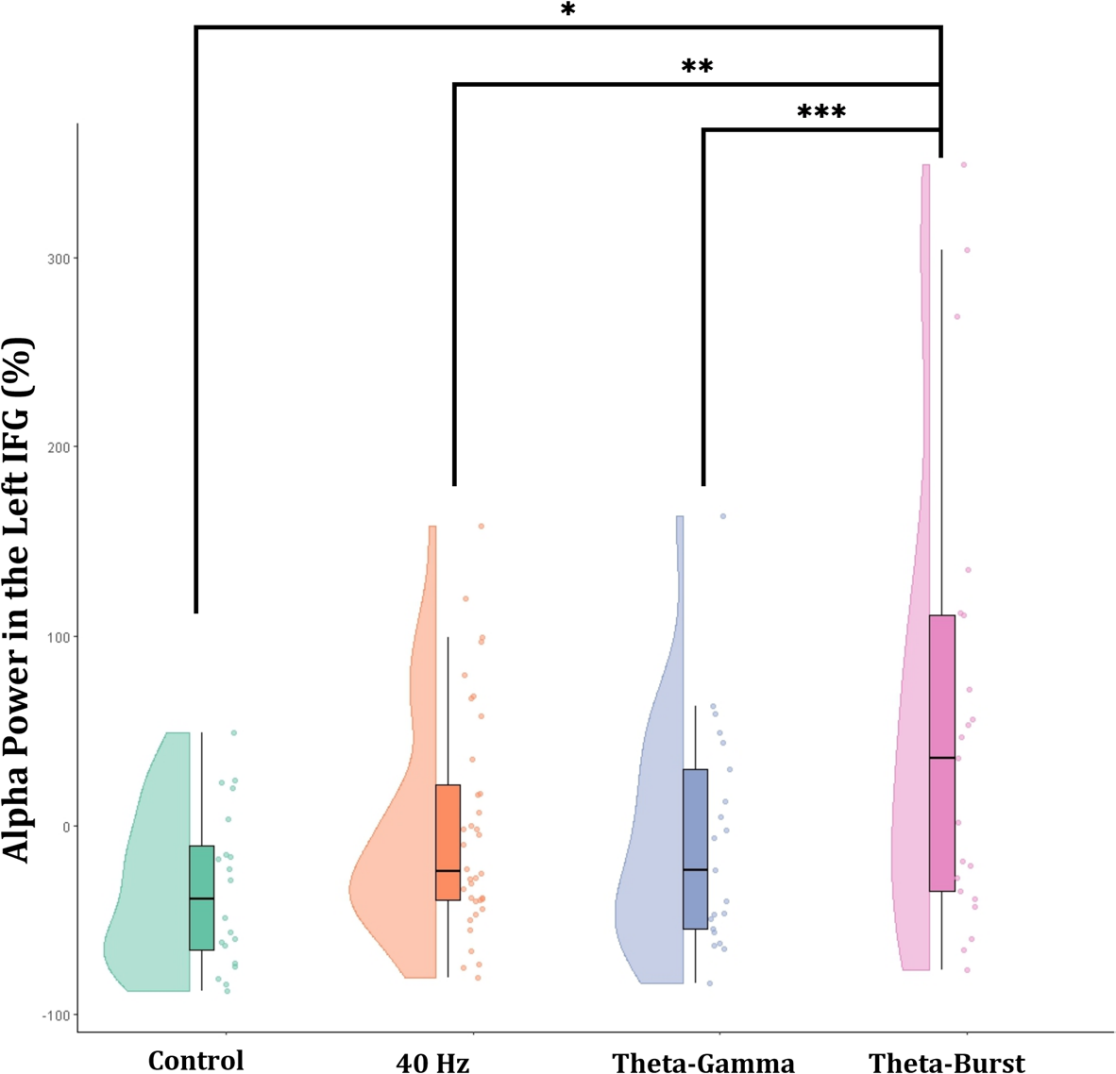
Raincloud plots of high alpha activity (10–12 Hz) in the left inferior frontal gyrus across EMF exposure patterns. Theta-Burst EMF significantly increased high alpha activity compared to the Control, 40 Hz, and Theta-Gamma condition. Raincloud plots display individual data points (right), boxplots indicating the interquartile range with whiskers representing the data range (center), and half-violin plots illustrating the distribution density (left). The asterisk (*) denotes a significant difference.

We conducted ROI-based ANOVAs on the bilateral DIPC and IFG as a priori working memory–related regions of interest, selected based on prior studies. A one-way ANOVA revealed a significant difference in the percentage change of high-alpha activity in the left IFG.[F(3,97) = 5.387, *p* = 0.002, eta^2^=0.15]. Tukey’s HSD confirmed that the Theta-Burst EMF condition exhibited significantly more high alpha activity than the Sham (*p* = 0.002, Cohen’s d = 0.888), 40 Hz (*p* = 0.012, Cohen’s d=-0.697), and Theta-Gamma condition (*p* = 0.020, Cohen’s d = 0.666) [Theta-Burst: 71.71(108.73) vs. Sham: 1.92(15.001), vs. 40 Hz 20.11(43.14), vs. Theta-Gamma: 16.94(41.08), Mean ± SD]. This result can be seen in Fig. 6. No significant effects were observed in the right IFG or in the DIPC bilaterally (*p* > 0.05). Finally, no significant interaction effects were observed between EMF placement condition and EMF type for either WAIS-IV or EEG measures (all *p* > 0.10). This suggests that the observed differences were driven primarily by the frequency characteristics of the EMF exposure rather than its spatial placement.

## Discussion

This study tested the hypothesis that Theta-Burst EMF exposure would impair working memory performance and explored whether Theta-Gamma and 40 Hz EMFs exerted similar effects on memory and neural dynamics. The results showed that Theta-Burst EMF significantly reduced working memory performance on the DSB subtest and was accompanied by increased high-alpha activity in the left IFG and a whole-brain sLORETA effect centered on the right superior frontal gyrus. In contrast, Theta-Gamma EMF reduced short-term recall on the DSF subtest without producing detectable EEG changes. These findings indicate that different EMF patterns exert distinct and pattern-specific behavioral and neural effects.

Theta-Burst EMF produced converging behavioral and EEG effects in frontal regions. The IFG high-alpha increase is often interpreted as a marker of functional inhibition, and prior studies have linked excess alpha synchronization in this region to weakened memory performance^8,9^. The right SFG sLORETA effect further implicates medial and superior frontal regions in the observed working memory impairment, consistent with the role of the fronto-parietal network in executive control and attentional regulation^45,47^. Beyond the frontal regions, no significant effects were observed in the bilateral DIPC, despite its established role in working memory maintenance and manipulation. This absence suggests that the Theta-Burst EMF effects observed here may be spatially constrained to frontal nodes of the network rather than engaging parietal components. Together, the frontal regional involvement and concurrent behavioral–neural changes align with prior reports of Theta-Burst stimulation altering network activity involved in cognitive control^29,35,36^. Given the low-density EEG configuration, source-projected results should be interpreted as regionally constrained summaries of cortical activity rather than precise anatomical localization.

Theta-Gamma EMF produced a distinct pattern, reducing DSF performance without measurable EEG changes. This could reflect more subtle or diffuse neural effects that fall below the detection threshold of the current analysis, or modulation of cognitive processes that are not strongly expressed in oscillatory measures captured here. Given that Theta-Gamma coupling is linked to hippocampal–cortical interactions during encoding, the observed behavioral effect may represent a targeted disruption of short-term storage processes rather than the manipulation demands seen in working memory tasks.

The difference between the DSF and DSB outcomes emphasizes the importance of subtest-specific demands. DSB requires active manipulation of information and engages executive control processes, while DSF largely measures passive retention. That Theta-Burst selectively impaired DSB, and Theta-Gamma selectively impaired DSF, suggests that EMF effects can target distinct cognitive operations depending on the stimulation pattern. Interestingly, neither effect was present in any WAIS-IV composite scores. Composite measures combine performance across multiple subtests with different cognitive and neural demands, which may recruit compensatory processes from unaffected domains, effectively masking targeted deficits. This supports the view that EMF-related modulation can be localized and functionally selective, and may be overlooked when using broad aggregated measures.

These results highlight the nuanced effects of different EMF patterns on cognitive and neural function. Theta-Burst EMF selectively disrupted working memory and was accompanied by frontal high-alpha increases in both the IFG and right SFG, whereas Theta-Gamma EMF primarily affected short-term recall without detectable EEG correlates. The absence of effects in the DIPC further suggests that these influences may be spatially specific rather than broadly distributed across the fronto-parietal network.

## Conclusion

This work adds to converging evidence that EMFs can act as structured influences on brain dynamics, with effects that depend strongly on stimulation pattern and frequency. The broader challenge now is to determine the precise conditions under which neuromodulation facilitates versus disrupts cognition, and to uncover the mechanisms by which these effects propagate through neural systems. Establishing these parameters will be essential for translating EMF-based approaches into reliable tools for cognitive enhancement, therapeutic intervention, or targeted modulation in neurological disorders.

## Supplementary Information

Below is the link to the electronic supplementary material.

Supplementary Material 1

## Data Availability

The data that support the findings of this study are not openly available due to reasons of sensitivity and are available from the corresponding author upon reasonable request.
