# Supplementary material for: Electromagnetic field stimulation modulates working memory and cortical alpha oscillations in healthy adults
Source: Sci Rep. 2026 Feb 26;16:8660. doi: 10.1038/s41598-026-42063-4 (PMC12979613; doi:10.1038/s41598-026-42063-4)
Supplement: Supplementary file 1 — Supplementary Material 1 [file 41598_2026_42063_MOESM1_ESM.docx]

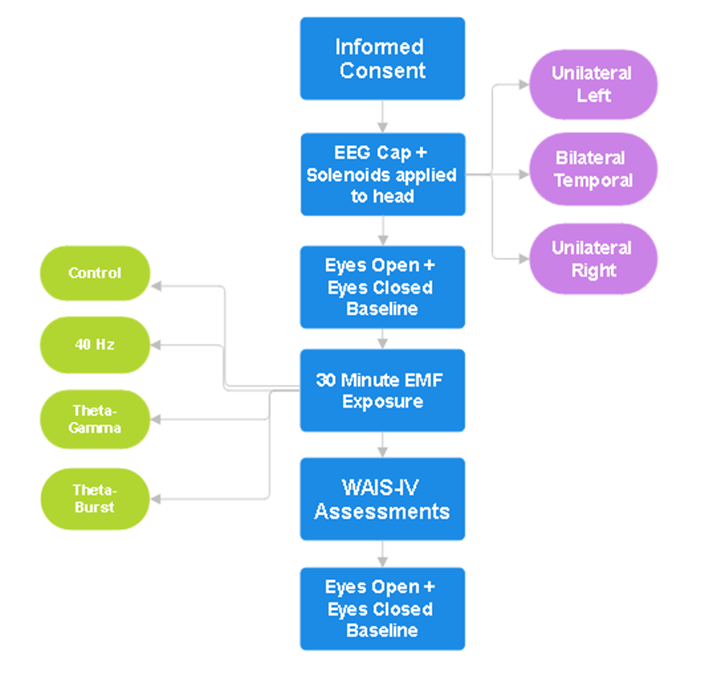


**Supplementary Figure S1. Experimental Procedure Flowchart:** Flow diagram illustrating the experimental procedure. After informed consent, participants were fitted with an EEG cap and solenoids. Baseline EEG recordings were collected (eyes open and closed). Participants were then randomized to one of four conditions (Control, 40 Hz, Theta-Gamma, or Theta-Burst) and exposed to a 30-minute EMF session with solenoids placed unilaterally left, unilaterally right, or bilaterally over temporal regions. Following exposure, WAIS-IV cognitive assessments were administered, and a second baseline EEG recording (eyes open and closed) was completed.
